# Who Are the Nonusers of Sunscreen, and What Are Their Reasons? Development of a New Item Set

**DOI:** 10.1007/s13187-020-01732-2

**Published:** 2020-03-06

**Authors:** Katharina Diehl, Sven Schneider, Svenja Seuffert, Rüdiger Greinert, Tatiana Görig

**Affiliations:** 1 Mannheim Institute of Public Health, Social and Preventive Medicine, Medical Faculty Mannheim, Heidelberg University, Ludolf-Krehl-Straße 7-11, 68167 Mannheim, Germany; 2 Association of Dermatological Prevention (ADP), Hamburg, Germany; 3 Center of Dermatology, Elbe Clinics, Buxtehude, Germany

**Keywords:** Skin cancer, Sunscreen, Barriers, Sun protection, Germany

## Abstract

Sunscreen use is an important aspect of sun protective behavior. Previous studies revealed deficits in sunscreen use. Our aim was to quantify sunscreen use in a nationwide representative study in Germany as well as to develop and test an item battery on reasons for none use of sunscreen. We analyzed data of the National Cancer Aid Monitoring (NCAM; wave 4; *n* = 3000, aged 14–45). To describe those who only use sunscreen rarely or never, we used chi^2^ statistics and logistic regression analysis. In addition, we utilized a newly developed item battery on barriers to sunscreen use. Here, we used Cronbach’s alpha to investigate reliability. In total, 20.7% reported using sunscreen rarely or never. Infrequent or none use of sunscreen was associated with male sex, immigrant background, none or rare sunbathing in summer, and current or past use of sunbeds. Participants with higher skin cancer risk (e.g., pale skin) were less likely to use sunscreen infrequently or never. The major reasons for not using sunscreen were inconvenience and no perceived need for applying sunscreen. Overall, internal consistency of the item battery on potential barriers to sunscreen use was very good (Cronbach’s alpha = 0.865). We found deficits in sunscreen use especially in sunbed users, men, and individuals with immigrant background. Our results give important implications for future prevention and health promotion campaigns on sunscreen use.

## Introduction

During the past decades, we observed a worldwide increasing incidence of skin cancer in the Caucasian population [1–3]. The main environmental risk factor for most common types of skin cancer such as basal cell carcinomas (BCC), squamous cell carcinomas (SCC), and malignant melanoma is ultraviolet (UV) radiation—specifically solar radiation [4, 5]. Therefore, one main aspect in skin cancer prevention is the promotion of sun protective behavior.

Sun protective behavior includes the use of sunscreen, wearing protective clothing, staying in the shade, and avoiding the outdoors during midday [6]. From previous studies, we know that there are deficits in sun protective behaviors [7–10]. Furthermore, we know that these behaviors are associated with sex, age, and education level [11–15]. The majority of previous studies conducted in this field only focused on specific subgroups (e.g., adolescents; [16]). In order to develop nationwide prevention programs, it would be necessary to have reliable representative data.

In wave 1 of the National Cancer Aid Monitoring (NCAM), we found that sunscreen use is less frequent than sun protective measures such as wearing sunglasses, staying in the shade, and wearing long-sleeved clothes [7]. Therefore, our first aim in wave 4 of the NCAM was to identify in detail population groups that use sunscreen rarely or never. In addition, to follow our second aim, we explored reasons for infrequent sunscreen use by developing a new item battery and tested it in our nationwide representative study. The identification of barriers for sunscreen use in our nationwide representative study is important for future prevention and health promotion programs that aim to decrease the incidence of skin cancer.

## Methods

### Study Setting and Study Sample

The data for this study were drawn from the National Cancer Aid Monitoring (NCAM). As part of this representative nationwide study on natural and artificial UV exposure, additional data on the use of sunscreen were collected in the fourth NCAM wave from October to December 2018 (response rate 28.5%). In total, 3000 individuals aged between 14 and 45 years were interviewed in standardized computer-assisted personal telephone interviews (CATI). Participants were selected randomly based on an established two-stage selection process. Detailed methods of NCAM have been described elsewhere [17]. All participants gave informed consent to participate in the study. The study was approved by the Ethics Committee of the Medical Faculty Mannheim, Heidelberg University (2007-269E-MA).

### Measures

#### Frequency of Sunscreen Use

Following Glanz et al. [18], participants were asked how often they use sunscreen with a sun protection factor (SPF) of 20 or higher when they are outdoors on a sunny summer day for more than 15 min (never, rarely, sometimes, often, always). To identify population groups of infrequent users of sunscreen, we dichotomized this variable into those who reported using sunscreen (sometimes, often, always) and those who reported infrequent or none use of sunscreen (never, rarely).

#### Sociodemographic Variables

We included information about sex (male, female), age (14–17 years, 18–25 years, 26–35 years, 36–45 years), immigrant background (no, yes), school education (low, medium, high), employment status (none, full-time/part-time), and having a partner (no, yes).

#### Skin Characteristics

Participants were asked about individual skin type following Fitzpatrick (skin type I or II vs. skin type III to VI), having sunburns before the age of 15 (rarely/do not know vs. often), more than 40 birthmarks (no/do not know vs. yes), a family history of malignant melanoma (no/do not know vs. yes), and a personal history of malignant melanoma (no/do not know vs. yes). All these characteristics are risk factors for developing skin cancer.

#### Tanning Behavior

Questions about the tanning behavior included asking participants about the frequency of sunbathing during the last summer (rarely/never, sometimes, very often/often) and sunbed use in general (never, past, current). Participants who reported using sunbeds at least once during the last 12 months were considered current users.

#### Reasons for Infrequent or None Use of Sunscreen

In the subgroup of participants who used sunscreen infrequently or never, we assessed agreement with 25 items on not using sunscreen (for an overview of all 25 items, see Table 2). The items were selected based on previous research [19–22]. In addition, they were tested in a comprehensive cognitive pretest (*n* = 15). Based on its results, wording of some items was altered and specified. Additional reasons for none or infrequent use were identified and integrated into the item battery (e.g., “because sunscreen gets into the water and damages the environment”). We made the decision to repeat the introduction “I do not use sunscreen because …” before every fifth item, to ensure that items are answered as potential reasons for none/infrequent use and are not understood as general items to answer. The 25 items were asked in random order.

### Statistical Analyses

First, we describe individuals, who infrequently or never use sunscreen in more detail, based on the abovementioned variables on sociodemographics, skin characteristics, and tanning behavior by using chi^2^ tests. Additionally, we calculated logistic regression models with variables that showed significant associations in bivariate analyses to identify the main characteristics associated with none or infrequent sunscreen use: sociodemographics (model I), skin characteristics (model II), and tanning behavior (model III). Model IV is the total model which includes all independent variables that were significant in models I to III.

Second, we analyze the item battery consisting of 25 potential reasons for none or infrequent use of sunscreen. After descriptive analysis including chi^2^ statistics, we calculated Cronbach’s alpha to assess internal consistency (reliability) of the item battery. To group the 25 items, we build six subcategories based on content. For each subcategory, we calculated sum scores of the items (yes = 1) and determined the mean. In addition, we compared means regarding sex, age group, and skin type. Since sum scores were not normally distributed, we respectively calculated Mann-Whitney *U* tests and Kruskal-Wallis *H* tests.

For all analyses, a *p* value of < 0.05 was defined as being significant a priori. To ensure the national representativeness of the sample, the data were weighted by age, sex, education, and federal state. All analyses were conducted using IBM SPSS Statistics 25 (IBM Corporation, Armonk, NY).

## Results

The mean age of the 3000 respondents was 30.2 years (SD = 9.0), and women represented 48.6% of the sample. The majority (80.8%) of all participants had no immigrant background and nearly 26% had a low school education (still at school, left school without certificate, or secondary modern school qualification). Thirty-six percent of respondents had very fair or fair skin (Fitzpatrick’s skin type I or II).

Regarding the use of sunscreen, the majority reported using sunscreen sometimes (33.0%), often (29.1%), or very often (17.3%) on a sunny summer day. About one-fifth used sunscreen rarely (12.3%) or never (8.4%).

### Determinants Associated with Infrequent or None Use of Sunscreen

Bivariate analyses and logistic regression analyses showed that the infrequent or none use of sunscreen is associated with sociodemographic variables (sex, age, immigrant background), skin characteristics (skin type, more than 40 birthmarks, family history of malignant melanoma), and tanning behavior (sunbathing in summer, sunbed use, Table 1).

**Table 1:** Logistic regression analyses on determinants associated with infrequent or none use of sunscreen

		Chi^2^ test		Logistic regression model							
		Infrequent or none use of sunscreen		Model I		Model II		Model III		Model IV	
		%	*p* value	OR	*p* value	OR	*p* value	OR	*p* value	OR	*p* value
Sociodemographic variables											
Sex			< 0.001								
	Male	24.7		1.000						1.000	
	Female	16.4		0.595	< 0.001					0.610	< 0.001
Age groups			< 0.001								
	14–17	22.0		1.000						1.000	
	18–25	18.6		0.804	0.227					0.713	0.057
	26–35	14.6		0.612	0.008					0.537	< 0.001
	36–45	28.0		1.471	0.034					1.101	0.567
Immigrant background			< 0.001								
	No	19.1		1.000						1.000	
	Yes	27.0		1.523	< 0.001					1.625	< 0.001
Education			0.010								
	Low	20.2		1.000							
	Medium	23.6		1.154	0.288						
	High	18.2		0.872	0.329						
Employment			0.591								
	None	21.5									
	Full-time/part-time	20.5									
Partnership			0.752								
	No	21.0									
	Yes	20.5									
Skin cancer risk											
Skin type			< 0.001								
	I/II	12.7				1.000				1.000	
	III–VI	24.9				2.192	< 0.001			2.127	< 0.001
Sunburn before the age of 15			0.327								
	Rarely/ Do not know	20.9									
	Often	18.1									
More than 40 birthmarks			< 0.001								
	No/do not know	22.9				1.000				1.000	
	Yes	15.7				0.690	< 0.001			0.644	< 0.001
Family history of malignant melanoma			0.002								
	No/do not know	21.4				1.000				1.000	
	Yes	13.5				0.609	0.006			0.587	0.004
History of malignant melanoma			0.371								
	No/do not know	20.8									
	Yes	16.9									
Tanning behavior											
Sunbathing in summer			< 0.001								
	Rarely/ never	28.6						1.000		1.000	
	Sometimes	12.9						0.373	< 0.001	0.358	< 0.001
	Very often/ often	19.3						0.594	< 0.001	0.550	< 0.001
Sunbed use			< 0.001								
	Never	18.4						1.000		1.000	
	Past	28.3						1.707	< 0.001	1.574	< 0.001
	Current	23.5						1.434	0.023	1.458	0.023
*n*				2726		2977		2977		2956	
*r*^2^_Nagelkerkes_				0.060		0.046		0.052		0.139	

Dependent variable: 1 = using sunscreen rarely or never, 0 = using sunscreen sometimes/often/very often

Models I–III: logistic regression models only included variables that were significant in bivariate analyses

Model IV: logistic regression model only included variables that were previously significant in models I–III

Data from the fourth wave of the National Cancer Aid Monitoring (NCAM)

*n* = 3000 individuals aged 14–45 years old living in Germany

Data weighted by age, sex, education, and federal state

*OR* odds ratio

Females used sunscreen less infrequently than males (16.4% vs. 24.7%; OR = 0.610, *p* < 0.001; model IV Table 1). Regarding age, we found a U-shaped association with infrequent or none use of sunscreen. Respondents with immigrant background were more likely to use sunscreen infrequently or not at all than their counterparts did (27.0% vs. 19.1%; OR = 1.625, *p* < 0.001; model IV). The same applies to participants with darker skin compared with those with pale skin (24.9% vs. 12.7%; OR = 2.127, *p* < 0.001). Individuals who have more than 40 birthmarks were less likely to use sunscreen infrequently or not at all than individuals who have less than 40 birthmarks (15.7% vs. 22.9%; OR = 0.644, *p* < 0.001). Respondents with a family history of malignant melanoma were less likely to use sunscreen infrequently than those without (13.5% vs. 21.4%; OR = 0.587, *p* = 0.004). Those who sunbathed rarely or never during the last summer were more likely to use sunscreen infrequently or not at all (28.6%; OR = 1.000) compared with those who sunbathed sometimes (12.9%; OR = 0.358, *p* < 0.001) or very often to often (19.3%, OR = 0.550, *p* < 0.001). Regarding sunbed use, we found past (28.3%, OR = 1.574, *p* < 0.001) and current users (23.5%; OR = 1.458, *p* = 0.023) being more likely to show none or an infrequent sunscreen use compared with never users (18.4%; OR = 1.000).

### Reasons for None or Infrequent Use of Sunscreen

In the subgroup of 617 participants who used sunscreen infrequently or never, the majority reported inconvenience (45.8%, *n* = 283) and no need for applying sunscreen (40.2%, *n* = 247) as reasons for not using sunscreen. Looking unattractive due to sunscreen (7.4%, *n* = 46), peer influence (“my friends don’t use sunscreen either”; 7.0%, *n* = 43), portrayal as an effeminate person due to sunscreen use (“using sunscreen makes me look weak”; 6.1%, *n* = 38), and the price of sunscreens (4.6%, *n* = 28) played a minor role as barriers for sunscreen use. Table 2 presents the 25 items regarding reasons for not using sunscreen and the subgroups’ agreement/disagreement with these items.

**Table 2:** Reasons for infrequent or none use of sunscreen

I do not use sunscreen because…	Agreement		Disagreement		Do not know	
	*n*	%	*n*	%	*n*	%
1. Lack of commitment						
… it is too inconvenient	283	45.8	331	53.7	3	0.5
… I am too lazy to use sunscreen	238	38.6	376	60.9	3	0.5
… I often forget to apply sunscreen	218	35.4	390	63.2	9	1.4
2. Lack of risk awareness						
… I do not need sunscreen	247	40.2	362	58.9	5	0.9
… sun protection is not important to me	208	33.8	400	65.0	7	1.2
… my skin is insensitive	147	23.9	461	74.8	8	1.3
… sunscreen prevents me from getting tanned	100	16.2	508	82.5	8	1.3
3. Barriers related to application of sunscreen						
… it bothers me that one has to reapply it	218	35.4	393	63.8	5	0.9
… it is often too stressful for me to wear sunscreen	173	28.1	436	70.7	7	1.2
...I cannot get to all parts of the body, like my back	141	22.9	470	76.6	3	0.5
… applying sunscreen takes too much time	105	17.1	504	82.1	5	0.8
… I have difficulties applying sunscreen	84	13.7	526	85.3	6	1.0
4. Unpleasant side-effects of sunscreen						
… sunscreen makes sand stick to the skin	190	30.8	419	68.0	7	1.2
… sunscreen gets into the water and damages the environment	105	17.1	495	80.3	16	2.6
… sunscreen burns the eyes	87	14.2	512	83.2	16	2.7
… I get pimples when I use sunscreen	87	14.2	518	84.1	11	1.7
… my skin is allergic to sunscreen	65	10.5	546	88.6	6	0.9
5. Peer group–related barriers						
… sunscreen on the skin makes me look unattractive	46	7.4	558	90.8	11	1.8
… my friends do not use sunscreen either	43	7.0	554	89.9	19	3.0
… using sunscreen makes me look weak	38	6.1	561	91.3	16	2.6
6. Barriers related to product characteristics						
… I find sunscreen too sticky or oily/greasy	223	36.2	388	62.9	6	1.0
… sunscreen leaves a white film on the skin	122	19.7	483	78.3	12	2.0
… sunscreen has an unpleasant odor	85	13.8	524	85.2	6	1.0
… the cream on skin shines too much	65	10.6	541	87.8	10	1.6
… sunscreen is too expensive	28	4.6	575	93.4	12	2.0

Data from the fourth wave of the National Cancer Aid Monitoring (NCAM)

*n* = 617 individuals who reported infrequent or none use of sunscreen

Data weighted by age, sex, education, and federal state

Overall, internal consistency of the item battery on potential barriers to sunscreen use was very good (Cronbach’s alpha = 0.865). In order to organize the 25 items, we grouped them into six categories driven by content. While we did not find any significant sex difference, we found differences in the importance of grouped reasons by age and skin type (Table 3). We found those aged between 18 and 35 years are more likely to report barriers related to the application of sunscreen (e.g., bothers by reapplication; *p* = 0.001) and product characteristics (e.g., stickiness; *p* = 0.011). Peer group–related barriers (e.g., sunscreen makes one look unattractive) were more important for those being aged 14 to 25 years (*p* = 0.001). Individuals with skin type I or II were more likely to report reasons regarding commitment (e.g., inconvenience) and application-related barriers (*p* = 0.015 and *p* = 0.006, respectively). Individuals with darker skin (i.e., skin type III or higher) were more likely to name lack of risk awareness (e.g., no need of sunscreen) and peer group–related reasons as barriers to sunscreen use (*p* < 0.001 and *p* = 0.006, respectively).

**Table 3:** Reasons for infrequent or none use of sunscreen by sex, age groups, and skin type

Reasons for infrequent or none use of sunscreen	Total			Sex			Age groups					Skin type		
	*n*	Mean (SD)	Min/max	Female	Male	*p* value	14–17	18–25	26–35	36–45	*p* value	I/II	III–VI	*p* value
1. Lack of commitment	617	1.20 (1.10)	0/3	1.15	1.23	0.312	1.07	1.32	1.33	1.11	0.117	1.38	1.15	0.015
2. Lack of risk awareness	611	1.14 (1.07)	0/4	1.06	1.19	0.261	1.03	1.22	1.21	1.09	0.544	0.75	1.24	< 0.001
3. Barriers related to application of sunscreen	611	1.17 (1.32)	0/5	1.12	1.20	0.439	1.05	1.39	1.46	0.94	0.001	1.33	1.13	0.006
4. Unpleasant side-effects of sunscreen	615	0.87 (1.06)	0/5	0.91	0.84	0.193	0.90	0.92	0.94	0.79	0.491	0.77	0.90	0.464
5. Peer group–related barriers	613	0.21 (0.53)	0/3	0.17	0.23	0.230	0.29	0.34	0.21	0.12	0.001	0.10	0.24	0.006
6. Barriers related to product characteristics	612	0.84 (1.07)	0/5	0.90	0.80	0.120	0.65	0.96	1.07	0.70	0.011	0.83	0.84	0.909

Dependent variable: sum score mean value of each subcategory

Data from the fourth wave of the National Cancer Aid Monitoring (NCAM)

Data weighted by age, sex, education, and federal state

*n* = 617 individuals who reported infrequent or none use of sunscreen

## Discussion

Within this manuscript, we followed two aims: First, we wanted to describe those who use sunscreen infrequently or not at all in more detail. Second, we aimed to test a newly developed item battery on barriers for none or infrequent use of sunscreen. We found that none or infrequent use of sunscreen is more likely among males, respondents with immigrant background, those who sunbathe irregularly in summer, and among sunbed users. Respondents with a higher skin cancer risk (i.e., skin type I or II, more than 40 birthmarks, family history of malignant melanoma) were more likely to use sunscreen. Most important reasons for not using sunscreen (regularly) were inconvenience and lack of perceived need to apply sunscreen.

### Use of Sunscreen by Individual Characteristics

Regarding the association of sunscreen use and sex, our results are consistent with previous studies: Females were more likely to use sunscreen [9, 23]. Furthermore, some studies showed that females are generally more likely to perform sun protective behavior (e.g., wearing sunglasses, wearing covering clothing, staying in the shade, applying sunscreen) than males [9, 24]. This may be explained by a higher general risk awareness among women [25, 26]. For age, we found a U-shaped association with sunscreen use. The high prevalence of none or infrequent use in minors may be responded by more education on UV-related issues in school. Regarding the difference in use between individuals with and without immigrant background, future research on the country of origin would be helpful to study this aspect in more detail. We also were not able to explore associations between use of sunscreen and marital status, although marriage can be a protective factor for melanoma incidence and mortality [27] and can foster sun protection at least in people aged 50 years and older [28]. Since we did ask our participants if they have a partner instead of asking if they are married, our analysis was based on a different concept.

In line with Basch et al. [23], we found that individuals with darker phenotypes are less likely to use sunscreen. Maybe they feel safe due to their darker natural skin type. However, this may lead to an overestimation of their skin’s self-protection against solar UV radiation, which in turn can increase the risk of sunburns and the development of melanoma and non-melanoma skin cancer. The finding that individuals with pale skin (skin type I and II) are more likely to use sunscreen goes well together with the finding that individuals with more than 40 birthmarks and a family history of malignant melanoma are more likely to use sunscreen. All these skin health characteristics were shown to be associated with higher skin cancer risk [29, 30]. Therefore, the majority of participants with these characteristics show an ideal behavior.

Regarding sunbed use, we found never users being more likely to use sunscreen compared with past and current users. This may also underline a general health-conscious behavior. Accordingly, sunbed users may face a double risk, which is important information for future prevention and potential extension of legislation regarding sunbed use. At the moment, unsupervised sunbeds are allowed and sunbed use is banned only for minors in Germany. We also found interesting results on sunbathing in summer: Those who rarely or never sunbathe showed the highest prevalence of none or infrequent use of sunscreen. This seems logically as they may not be in situations where they need sunscreen. Those who sunbathe (very) often seem to be a problematic group. Their likelihood to never use sunscreen or to do it infrequently (19.3%) was higher than in those who sunbathe sometimes (12.9%). This may be an indicator for less risk awareness. Maybe these individuals underlie a popular misconception that sunscreen prevents from getting tanned and therefore sunscreen is intentionally neglected to get a deep tan faster. Compatible with this, Robinson [31] showed that sun protection was not always perceived as a benefit because of the reduction of the tanning aspect.

### Reasons for Infrequent or None Use of Sunscreen

Most important reasons for not using sunscreen (regularly) describe inconvenience, perceived lack of need, and perceived unnecessity. Benvenuto-Andrade et al. [8] found impatience in applying sunscreen as a major obstacle for rare applications. Studies from other countries showed lack of time and forgetfulness as reasons for not using sunscreen [20, 32, 33]. Studies from Australia found greasiness and the need to reapply sunscreen products [21], as well as shiny looking and undesirable smell of sunscreen [22], as barriers to the use of sunscreen. However, most of these previous studies did not use an item battery as comprehensive as ours. Some used open-ended questions [8, 22, 33], which may lead to a recall bias and respondents might forget to mention reasons. In addition, these former studies did not analyze large representative samples.

Our results give important implications for future prevention as well as for the sunscreen industry. Since inconvenience is a main barrier for sunscreen use—especially among those with skin type I or II—it seems important that more product innovations are developed and highlighted in advertisement, too. For instance, there are sunscreen sprays that are absorbed very fast but many individuals may link sunscreen to sticky or oily creams that last on the skin. This would also solve the problem some individuals have with the product characteristics. In addition to that, general education campaigns may be helpful to convince especially those with darker skin types that the use of sunscreen is important. Further education may also reduce peer group–related barriers for use.

### Strength and Limitations

The present study was the first to explore barriers to sunscreen use in a large and representative sample. We assessed and validated a new item battery on barriers to the use of sunscreen. In addition, we could describe those who use sunscreen infrequently or never in more detail. Our results are important for health authorities and future prevention and health promotion campaigns.

Nevertheless, some potential limitations of this study should be taken into account. First, this study is based on self-reported data. Therefore, we cannot entirely rule out a recall bias or tendency to respond to social desirability. This especially applies to the frequency of sunscreen use during last summer. To reduce this bias, we conducted standardized interviews via telephone with trained interviewers. For individual barriers to regular sunscreen use, the limitation of self-reported data is negligible. Second, we did not assess any further sun protection behavior than the use of sunscreen in wave 4. For example, some people can protect themselves by covering their skin with clothing or avoiding sun at noon. However, in wave 1 of NCAM, we found that especially the use of sunscreen is deficient [7] and needs more attention in epidemiologic studies. Therefore, we focused on this specific sun protection behavior in wave 4. Third, due to our cross-sectional study design, we cannot draw conclusions on causality of identified associations. Nonetheless, we are able to describe the status quo, which was the primary objective in our study.

## Conclusion

In summary, in our study, we could test a newly developed, comprehensive item battery on reasons for not using sunscreen. The results show that the item battery’s internal consistency is very good. In future studies, our item battery should be further validated in different subgroups. Furthermore, we revealed deficits of sunscreen use in specific subgroups such as sunbed users, males, and individuals with immigrant background. Here, future prevention and health promotion campaigns should specifically focus on these groups in order to decrease their risk of skin cancer.
